# Hepatitis A Virus Strains Circulating in the Campania Region (2015–2018) Assessed through Bivalve Biomonitoring and Environmental Surveillance

**DOI:** 10.3390/v13010016

**Published:** 2020-12-23

**Authors:** Giuseppina La Rosa, Pamela Mancini, Giusy Bonanno Ferraro, Marcello Iaconelli, Carolina Veneri, Rosa Paradiso, Dario De Medici, Teresa Vicenza, Yolande Therese Rose Proroga, Orlandina Di Maro, Anna Rita Ciccaglione, Roberto Bruni, Michele Equestre, Stefania Taffon, Angela Costantino, Maurizio Della Rotonda, Elisabetta Suffredini

**Affiliations:** 1 Department of Environment and Health, Istituto Superiore di Sanità, 00161 Rome, Italy; giuseppina.larosa@iss.it (G.L.R.); pamela.mancini@iss.it (P.M.); giusy.bonannoferraro@iss.it (G.B.F.); marcello.iaconelli@iss.it (M.I.); carolina.veneri@iss.it (C.V.); rosa.paradiso@iss.it (R.P.); 2 Department of Food Safety, Nutrition and Veterinary Public Health, Istituto Superiore di Sanità, 00161 Rome, Italy; dario.demedici@iss.it (D.D.M.); teresa.vicenza@iss.it (T.V.); 3 Department of Food Microbiology, Istituto Zooprofilattico Sperimentale del Mezzogiorno, 80055 Portici, Italy; yolande.proroga@cert.izsmportici.it (Y.T.R.P.); orlandina.dimaro@izsmportici.it (O.D.M.); 4 Department of Infectious Diseases, NRL for Human Viral Hepatitis, Istituto Superiore di Sanità, 00161 Rome, Italy; annarita.ciccaglione@iss.it (A.R.C.); roberto.bruni@iss.it (R.B.); michele.equestre@iss.it (M.E.); stefania.taffon@iss.it (S.T.); angela.costantino@iss.it (A.C.); 5 Executive Task Force Prevention and Veterinary Public Health, Region Campania, 80132 Naples, Italy; mdellarotonda@hotmail.com

**Keywords:** hepatitis A, HAV, sewage, bivalves, PCR, sequencing, typing

## Abstract

The genetic diversity of Hepatitis A Virus (HAV) circulating in the Campania Region in years 2015–2018 was investigated through the monitoring of sentinel bivalve shellfish and water matrices. Overall, 463 water samples (71 sewage samples, 353 coastal discharge waters, and 39 seawaters samples), and 746 bivalve shellfish samples were analyzed. Positivity for HAV was detected in 20/71 sewage samples, 14/353 coastal discharge waters, 5/39 seawaters, and 102/746 bivalve shellfish. Sixty-one of the positive samples were successfully sequenced and were characterized as genotype IA (*n* = 50) and IB (*n* = 11). The prevalent strain circulating in 2015 in both bivalves and waters was the IA strain responsible for the outbreak occurring around the same time in the Naples area. This variant was no longer identified in subsequent years (2017–2018) when, instead, appeared two of the IA variants of the multistate outbreak affecting men who have sex with men (MSM), VRD_521_2016, and RIVM-HAV16–090, with the former prevailing in both shellfish and water environments. HAV IB isolates were detected over the years in shellfish and in water matrices, but not in clinical samples, suggesting that this genotype had been circulating silently. An integrated surveillance system (environment/food/clinical cases) can be a useful tool to monitor changes in viral variants in the population, as well as an early warning system.

## 1. Introduction

Hepatitis A is an acute self-limiting illness, associated with fever, malaise, nausea, anorexia, and jaundice. Disease is caused by hepatitis A virus (HAV), a non-enveloped RNA virus belonging to the genus Hepatovirus of the family Picornaviridae [1,2]. HAV is classified into six genotypes (I–VI), in turn divided into subtypes, of which genotypes I to III can infect humans. Subtype IA is the most common HAV type worldwide (https://www.ecdc.europa.eu/en/hepatitis-A/facts).

Hepatitis A virus is responsible for a considerable number of viral hepatitis cases annually worldwide, the level of prevalence depending on socio-economic status conditions [3]. Different countries are characterized by either high, intermediate, or low HAV endemicity. In developing countries (such as in Asia, Africa, and Central/South America) HAV infection is highly endemic and most persons become infected (often in an asymptomatic way) in early childhood, with consequent overall low rates of reported disease and outbreaks [4,5]. Otherwise, developed countries (North America, Australia, Japan, and countries in Western/Northern Europe) display intermediate or low endemicity. Infection in children is sporadic due to high hygienic conditions and sanitation, but the virus can be introduced through person-to-person contact or through contaminated water and food, causing outbreaks in the adult population [6]. Moreover, transmission among travelers to endemic regions, sexual transmission in men who have sex with men (MSM), and transmission among people who inject drugs have also been reported [3].

Hepatitis A is a notifiable disease in Italy and, over the past few decades, the country has shifted from high to low/intermediate endemicity [7]. Within this period, hepatitis A incidence has decreased from 19 per 100,000 inhabitants in 1997, to 1.5 per 100,000 inhabitants in 2018 (https://www.epicentro.iss.it/epatite/bollettino/Bollettino-4-marzo-2019.pdf). However, hepatitis A outbreaks have been occasionally reported in association with consumption of contaminated frozen mixed berries (2013) [8,9,10] or bivalve shellfish (2004, 2008, and 2015) [11,12], or due to sexual transmission (2016–2017) [13,14,15,16].

In February 2015, sporadic cases of hepatitis A, potentially associated with food consumption, were reported in the Naples area (Campania Region). Upon request of the Regional Authority, the Italian Ministry of Health set up a task force composed of experts from the Ministry, the National Institute of Health (ISS, Rome), and the Istituto Zooprofilattico del Mezzogiorno (Portici, Naples), with the aim of identifying the possible source of contamination and adopting integrated control strategies. Through a combined human/food/environmental surveillance system embracing the One Health approach, HAV sequences obtained from human cases, bivalve shellfish, and water environments (sewage discharges and bivalve harvesting waters) were collected. Molecular data supplied by the integrated surveillance provided strong support for the adoption of focused control measures on bivalve production areas, allowing for containment of the outbreak, with the overall number of reported HAV cases held to less than a hundred in the peak period between January and June 2015, and with the complete restoring of water quality in shellfish production areas by the end of May 2015 [17]. When the emergency was concluded, the monitoring of water and shellfish samples continued, serving both as an early warning system and for epidemiological purposes.

In the study period another outbreak occurred (2016–2018), this time involving multiple states, affecting men having sex with men (MSM). The strains were first reported among MSM in the Netherlands (RIVM-HAV16-090, October 2016), England (VRD_521_206, December 2016), and Germany (V16-25801, January 2017) and were in the end identified in 22 European countries [18]. Between 1 June 2016 and 7 September 2018, 4475 cases were associated with this multi-country hepatitis A outbreak [19].

In this study, the diversity of HAV strains detected between 2015 and 2018 in bivalve shellfish and water environments in the Campania Region was assessed and compared with clinical strains identified by the National Reference Laboratory for Viral Hepatitis at the Department of Infectious Diseases of the ISS in order to determine the potential for an integrated molecular surveillance system.

## 2. Materials and Methods

### 2.1. Water Samples

**Figure 1:**
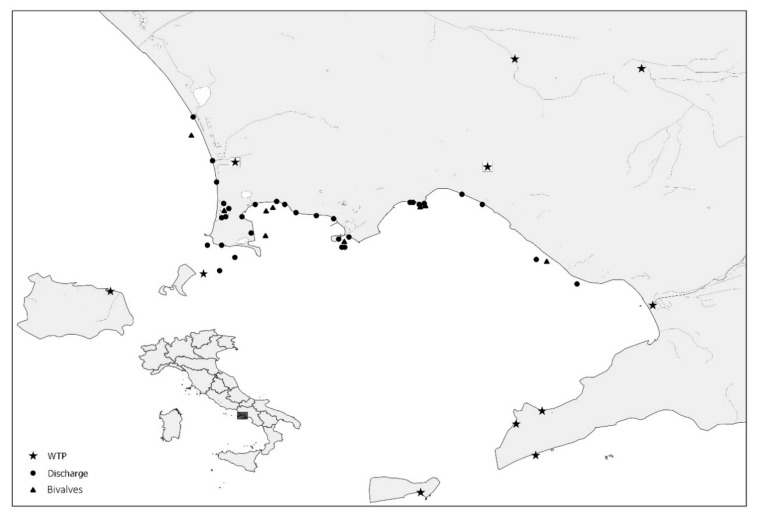
Geolocation of collection sites for water and bivalve samples.

A total of 463 water samples (71 sewage samples, 353 discharge water samples, and 39 seawater samples) were collected during the four years of the study. Ninety-eight samples were collected by the Campania Region’s Environmental Protection Agency (ARPA Campania) during the 2015 HAV outbreak presumptively associated with bivalve shellfish. In detail, 39 seawater samples were taken from shellfish growing areas (20 L each), 29 from nearby coastal discharge points (20 L each), and 30 raw sewage samples (500 mL each) from the eleven Wastewater Treatment Plants (WTPs) in the Campania Region. Subsequently, 365 samples were collected between 2017 and 2018 as follows: 324 samples were taken bi-monthly from the same discharge points along the coast monitored in 2015 and a further 41 sewage samples were collected from the two major WTPs in the region. Figure 1 shows the GIS (Geographic Information System) map of the collection sites included in the study.

Sewage samples were concentrated with a two-phase (polyethylene glycol/dextran) separation method [20]. For seawaters and discharge waters, concentration was performed using the adsorption–elution procedure. Mengovirus MC_0_ was used as a sample process control and the recovery rate was calculated as previously described [21]. For marine water samples, before concentration, the pH was adjusted to 3.5 with H_2_SO_4_; then, 20 L of water was filtered through a standard filter apparatus containing a sterile electronegative filter (Sartorius Membrane Filter Cellulose Nitrate 11306-142-G), using a peristaltic pump and a flow rate of 0.5 L/min. Then, 50 mL of 3% beef extract pH 9.5 was recirculated through the filters for 20 min for virus elution, and the pH was neutralized with HCl 1N. PEG 6000 and NaCl were added to reach final concentrations of 10% and 1.6% *w*/*v*, respectively, for the secondary concentration. The mix was incubated at 4 °C for 14–18 h and then centrifuged at 7000× *g* for 30 min at 4 °C. The supernatant was discarded and the pellet dissolved in 10 mL PBS pH 7.4. Discharge waters were concentrated as seawaters, with the difference that electropositive filters (3M Zeta Plus 1MDS) were used, which do not need the lowering of pH before concentration.

The concentrate was divided into two aliquots of 5 mL, one of which was immediately subjected to genome extraction, the other stored at −80 °C. Nucleic acid extraction and purification were performed using the NucliSens extraction kit and the MiniMag semi-automatic platform (bioMerieux, Paris, France) as described in the manufacturer’s instructions with minor modifications to adapt to large volumes (lysis buffer equivalent to twice the volume of the sample and lysis phase prolonged to 20 min). Eluted RNA (100 μL) was stored at −80 °C until molecular analysis.

### 2.2. Shellfish Samples

A total of 746 samples, including four bivalve mollusc species (*Mytilus galloprovincialis*, *Solen vagina*, *Venus gallina*, and *Donax trunculus*), were taken over 2015–2018 from 27 production areas of the coast and from biomonitoring points. In detail, 398 samples were taken during the 2015 HAV outbreak involving the Campania Region (February to August), 87 from September to December 2015, and the remaining 261 samples were collected periodically between 2016 and 2018. Analysis was performed according to ISO 15216-1, revision 2013 or 2017 based on processing year [22,23]. Briefly, depending on species’ size, 10 to 60 individuals were randomly selected and digestive tissue was dissected and finely chopped. Two-gram aliquots were spiked with 10 μL of Mengovirus MC_0_ process control (1.6 × 10^5^ TCID_50_/_mL_), digested with 2 mL of proteinase K (0.1 mg/mL; 37 °C for 60 min with shaking), and then placed at 60 °C for 15 min. Finally, the sample supernatant was collected after centrifugation at 3000× *g* for 5 min, and its volume was recorded. Nucleic acid extraction and purification were performed on 500 μL of supernatant using the NucliSens extraction kit (bioMerieux, France) according to the manufacturer’s instructions, and RNA was eluted in 100 μL and stored at −80 °C until molecular analysis.

### 2.3. Real Time PCR and Nested RT-PCR

Water samples underwent amplification by RT-nested-PCR using generic primers targeting the VP1/2A junction [24]. RNA (2 μL) and 10 pmol of forward and reverse primers were used in the first PCR, in a final mixture of 25 μL, using the MyTaq One Step RT-PCR kit (Bioline Ltd., London, UK). Amplification conditions were as follows: RT at 42 °C for 30 min, followed by RT at 95 °C for 2 min, 35 amplification cycles of denaturation for 30 s at 95 °C, annealing at 58 °C for 30 s, and extension at 72 °C for 1 min. Final incubation was performed at 72 °C for 10 min. Nested PCR was then performed with the MyTaq Red Mix (Bioline Ltd., London, UK), using 1 μL of the PCR product from the first step as a template. The nested PCR reaction conditions were 95 °C for 2 min, followed by 35 amplification cycles of 30 s at 95 °C, annealing at 50 °C for 30 s, and extension at 72 °C for 1 min.

Shellfish samples underwent an RT-PCR analysis for screening purposes, and then positive samples were subjected to the nested RT-PCR VP1/2A assay used for environmental samples. For RT-PCR analysis, the amplification conditions, primers, probes, and reagents were those reported in the annexes of the ISO 15216 method and previously published in separate papers [21,25]. Primers and probes used in this study are reported in Table 1.

Standard precautions were adopted in order to prevent contamination during PCR setup and PCR analysis. PCR products were analyzed by gel electrophoresis (2% agarose) and were visualized under UV light after staining with Gel Red (Biotium, Fremont, CA, USA). Positive PCR products were purified using a Montage PCRm96 Micro-well Filter Plate (Millipore, Burlington, MA, USA) and were sequenced directly on both strands (Bio-Fab Research s.r.l., Rome, Italy). The sequences were compared with HAV sequences deposited in GenBank using the BLAST program, as well as in the HAVNet (https://www.rivm.nl/en/havnet). The phylogenetic tree was constructed by the Neighbor-Joining algorithm using MEGA version X. Sequences were submitted to GenBank under the accession numbers MW279485–MW279545 (shellfish and water samples) and MW373449–MW373469 (clinical samples).

## 3. Results

Positivity for HAV RNA (Table 2) was detected in 39/463 water samples (20/71 sewage samples, 14/353 discharge waters and 5/39 seawaters). Upon analysis, the viral sequences were found to belong to genotype IA (*n* = 31) and IB (*n* = 8). Thirteen of the 39 positive samples were collected during the 2015 outbreak; of these, 10 were characterized as IA strains, showing 99–100% nt identity with the epidemic strain (ISS 1 1N 2015 Hu) isolated from patients with acute hepatitis, collected in the same region. In three samples (from sewage, discharge waters, and seawater, respectively), the virus was characterized as IB. Twenty-six of the 39 positive samples were instead collected in the period 2016–2018 and were characterized as IA (21 strains) and IB (5 strains). Of these IA strains, 11 were identical or highly similar to the variant RIVM-HAV16-090, and 10 showed 99–100% nt identity with the variant VRD-521-2016, both circulating during the multi-country hepatitis A outbreak among men who have sex with men (MSM). Only five IB strains were detected in 2017–2018, all very similar or identical to those detected in 2015.

A total of 102/746 shellfish samples tested positive for HAV by RT-qPCR; 77 of these had been collected during the 2015 outbreak period (February to August), 2 from September to December 2015, and the remaining 23 were distributed along the period from January 2016 to December 2018. Amplification by nested PCR was obtained in 22 samples, which were characterized by sequencing. These samples belonged to genotype IA (*n* = 19) and IB (*n* = 3). Most of the IA strains collected in 2015 (seven out of nine) showed 99–100% nt identity with the epidemic strain responsible for the local outbreak and with water samples collected in the same period. On the other hand, the IA strains collected in 2017–2018 (*n* = 10) showed 99–100% nt identity with the MSM variant VRD-521-2016. The RIVM-HAV16-090 and V16-25801 variants were not detected in shellfish samples. In three samples collected in 2015 (*n* = 2) and in 2018 (*n* = 1), the virus was characterized as IB.

**Figure 2:**
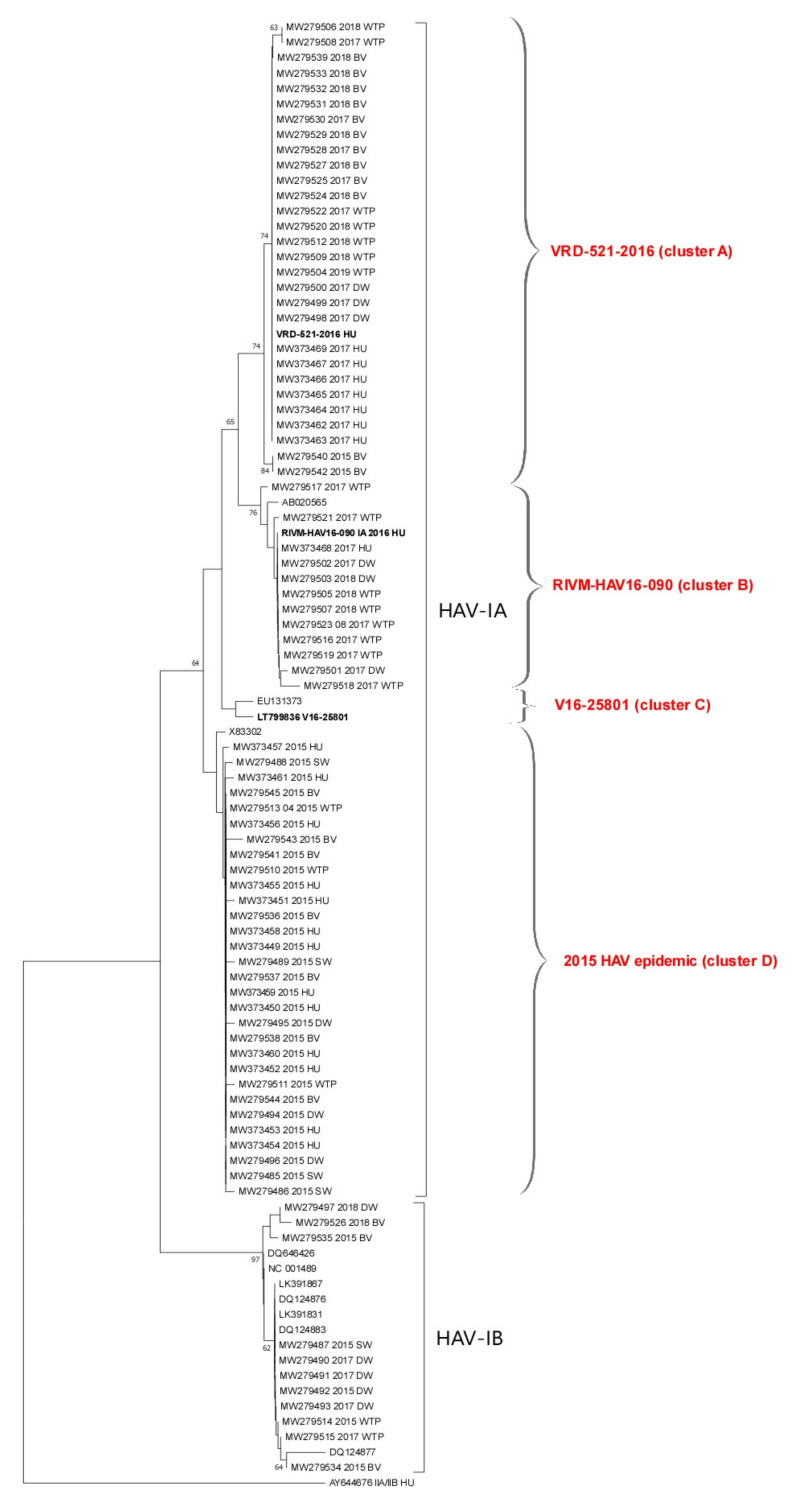
Phylogenetic tree of the hepatitis A virus VP1-2A region. The evolutionary history was inferred using the Neighbor-Joining method. The percentage of replicate trees in which the associated taxa clustered together in the bootstrap test (1000 replicates) is shown next to the branches (only bootstrap > 60% are shown). The tree is drawn to scale, with branch lengths in the same units as those of the evolutionary distances used to infer the phylogenetic tree. The evolutionary distances were computed using the Maximum Composite Likelihood method and are in the units of the number of base substitutions per site. This analysis involved 96 nucleotide sequences. There was a total of 197 positions in the final dataset. Analyses were conducted in MEGA X.

The results of the phylogenetic analysis are presented in Figure 2. The tree includes 61 study sequences: 20 sewage samples (suffix “WTP”), 14 discharge waters nearby shellfish growing areas (“DW”), 5 seawaters in production areas (“SW”), and 22 bivalve shellfish (“BV”). Twenty-three sequences obtained from serum samples of patients with acute hepatitis from the Campania Region in years 2015–2018, sequenced at the National Reference Laboratory for Viral Hepatitis (ISS, Rome), were also included in the tree. Finally, the tree includes prototype sequences of the multistate outbreak affecting MSM (RIVM-HAV16-090, VRD-521-2016, and V16-25801), as well as reference sequences from the HAVNet and GenBank and the outgroup sequence AY644676. Sequences from the present study are shown as ID code followed by year of sample collection and suffix.

Study sequences grouped into two main clusters corresponding to genotypes IA (78 sequences) and IB (18 sequences). The IA cluster was divided into four clusters, three of which related to the HAV genotype IA strains VRD-521-2016, RIVM-HAV16-090, and V16-25801 (clusters A, B, and C, respectively) responsible for the MSM outbreak, and one related to strains collected in 2015 (cluster D), containing highly related human (2015 outbreak), shellfish, and water sequences. Cluster A (VRD-521-2016 like) contains human, water, and shellfish isolates collected in 2017–2018 showing 100% nt identity among each other. Additionally, two IA strains collected in 2015 were grouped in this cluster. Cluster B (RIVM-HAV16-090-like) includes identical human and water samples collected between 2017 and 2019. None of the study samples clustered within the V16-25801 group. The HAV-IB cluster contains 11 water and shellfish samples, collected in 2015–2018. No clinical samples from the Campania Region clustered in the IB cluster. Some IB strains from sewage and discharge were identical to sequences previously found in urban sewages in Italy in 2012 (a.n. LK391831 and LK391867) [9], and to strains detected in 2004 in Italy (a.n. DQ124877) during an outbreak of hepatitis A virus infection with a high case-fatality rate among injecting drug users [26], therefore highlighting persistent circulation of this strain in the population in Italy.

## 4. Discussion

The aim of this study was to investigate the genetic diversity of HAV strains circulating in the Campania Region during 2015–2018 through the monitoring of water matrices and sentinel bivalve shellfish, and further comparison with clinical samples collected in the region in the same period. The integration of clinical, food, and environmental data represents the One Health approach of this study.

HAV is primarily transmitted from person to person via the fecal–oral route and through contaminated water and food such as shellfish and uncooked vegetables or fruit. The virus is shed in high numbers in the feces of infected individuals, reaching raw sewage in large quantities [27]. Due to high infectivity and resistance to wastewater treatments, the virus can contaminate surface waters used for different purposes, from recreational to drinking or irrigation [28].

The virus is present worldwide, and the level of seroprevalence depends on local sanitary conditions [29,30]. Hepatitis A is highly endemic in developing countries such as in Africa, Asia, South and Central America, and Eastern Europe, and displays an intermediate/low endemicity in developed countries such as North America, in Northern and Western Europe, and Australia [31,32]. Fulminant hepatitis is rare (<1%). The infection never becomes chronic, and clinical recovery results in immunity for life [3].

Studies from different countries have reported the presence of HAV in water matrices [33,34,35,36,37,38,39]. HAV occurrence has also been reported in foods [40,41,42,43,44] and in particular in onion [45], dried tomatoes [46], frozen berries [47], fresh vegetables [48,49], fruit juices [50], and shellfish [17,51,52,53,54,55,56,57,58,59,60,61,62]. Shellfish represent important vectors of viral illness due to their peculiar filtering capacity [63], an attribute that is also exploited for biomonitoring purposes [64,65,66].

In Italy, Hepatitis A is a notifiable disease with a low/intermediate endemicity and the outbreaks that occurred in the last few years were associated with foodborne and/or sexual transmission among MSM [8,9,10,11,13,14,15,16]. In 2013, an outbreak associated with consumption of frozen berries was reported in northern Italy (Emilia Romagna, Lombardy, Friuli Venetia Giulia, Trentino-Alto Adige, Piedmont, and Veneto) and southern Italy (Apulia) [9,10], and the responsible strain (genotype IA) was detected also in several European countries (Ireland, Holland, France, Great Britain, and Sweden). Therefore, the epidemic was classified as “multistate” [10]. About 1800 cases were documented during the outbreak and an environmental study, conducted on raw sewage collected during the epidemic period in different regions, showed that the hepatitis A virus was present in 24% of the samples (percentage according to a low/intermediate endemicity), including the IA variant responsible for the outbreak [9]. Two years later, an HAV outbreak, potentially associated with shellfish consumption, affected the Campania Region (south-central Italy). The multidisciplinary investigation identified another IA variant as responsible for the outbreak, and the same genotype was detected in shellfish, water, and serum samples from HAV-positive patients. Indeed, the phylogenetic tree of Figure 2 shows a genotype IA cluster (cluster D) containing 33 highly related sequences from human, shellfish, and water samples, all collected in 2015. In particular, HAV was detected in raw sewage samples, coastal discharge samples, and seawater samples. Most of the environmental sequences showed high nt identity with the IA strains isolated in the same geographic area from shellfish samples and from clinical cases notified during the ongoing outbreak. Two highly divergent IA strains also sampled in 2015 grouped instead in cluster A. Some strains from environmental samples collected in 2015, unrelated to the outbreak, were characterized as IB strains, and were identical to sequences previously found in urban sewages in Italy in 2012 (a.n. LK391831 and LK391867) [9] and to strains detected in 2004 in Italy (DQ124877) during an outbreak of hepatitis A virus infection with a high case-fatality rate among injecting drug users [26]. This suggests that the same strain had been circulating in Italy for years, or had been reintroduced some years later [11]. Significantly, within a few days of the first evidence of a rising outbreak, the multidisciplinary One Health approach adopted for the investigation demonstrated viral sequence identity among water, shellfish, and clinical sequences, providing the scientific evidence needed to implement significant control measures, and to limit to less than a hundred the HAV-notified cases in the observed period. It is noteworthy to mention that the case-count in previous seafood-associated outbreaks in Italy reached a considerably higher number of cases (5620 in 1996 in Apulia; 882 in 2004 in Campania) [67,68].

Since February 2016, another hepatitis A epidemic affecting MSM occurred in several European countries: Austria, Belgium, Denmark, Finland, France, Germany, Greece, Ireland, Italy, Malta, the Netherlands, Norway, Portugal, Slovenia, Spain, Sweden, and the United Kingdom. Three HAV genotype IA strains (VRD-521-2016, RIVM-HAV16-090, and V16-25801) were responsible for the outbreak [69]. Italy was among the most affected countries and from 1 January to 22 November 2017, 2583 cases of hepatitis A were reported [69,70,71,72]. In this study, the IA epidemic strain VRD-521-2016 of the multistate outbreak affecting MSM was found in human samples, different water matrixes, and shellfish collected in 2017–2018, while the strain RIVM-HAV16-090 was detected in both human samples and waters, but not in shellfish samples. The strain V16-25801 was not detected in field samples. In the same period, HAV detected in a few water and shellfish samples was characterized as IB. None of the clinical strains of the Campania Region clustered in the IB group, suggesting that this genotype had been circulating silently, causing asymptomatic infections, or that symptomatic clinical cases may have gone undetected to the surveillance system, because of under-reporting of infections.

In conclusion, the prevalent strain circulating in 2015 in both bivalves and waters was the IA strain responsible for the HAV outbreak occurring around the same time in Naples area. This variant was no longer identified in subsequent years (2017–2018) when, instead, appeared two HAV-IA variants of the multistate outbreak affecting MSM, VRD 521_2016 and RIVM-HAV16–090, with the former prevailing in both shellfish and water environments.

The present study demonstrates that a combined environmental/food/clinical surveillance system is able to provide a more complete picture of the circulation of HAV and of the prevalent genotypes in a community, allowing for a better understanding of changes in disease trends. Indeed, analysis of urban wastewaters [73] and of natural bio-concentrators such as bivalve shellfish [74] has been previously demonstrated to be an efficient approach for surveillance of human viruses as it provides information on their circulation in large shares of the population irrespective of clinical symptomatology and disease manifestation (the so-called ‘surveillance pyramid’). By monitoring the community, this approach provides a sensitive tool for the detection of the spread in the population of new viruses and of new viral variants, for the detection of viruses/variants with low diffusion, and for the study of seasonal and geographical trends of circulation. As such, environmental/food surveillance can provide both early warning signals and strategic data that can be used to (i) support focused public health actions (such as specific restrictions, information campaigns, etc.), and (ii) increase the efficacy of monitoring activities (including clinical surveillance) by pointing out areas of criticality. A full integration of environmental/food and clinical surveillance (i.e., complementation of monitoring on the community and on individuals) can therefore increase the overall sensitivity and efficiency of surveillance systems, and contribute to scientifically supported public health decision processes.

**Other members of the NRL for Human Viral Hepatitis:**
  - Michele Equestre—Department of Infectious Diseases, Istituto Superiore di Sanità, Rome, Italy
  - Stefania Taffon—Department of Infectious Diseases, Istituto Superiore di Sanità, Rome, Italy
  - Angela Costantino—Department of Infectious Diseases, Istituto Superiore di Sanità, Rome, Italy

## Figures and Tables

**Table 1:** RT-PCR and RT-qPCR primers and probes used in this study.

PCR	Primer Name	Primer Sequence (5′–3′)	Length (bp)	PCR Round	Reference
Nested-PCR	2897	TATTCAGATTGCAAATTAYAAT	393	1st round	[24]
	3288	AAYTTCATYATTTCATGCTCCT			
	2949	TATTTGTCTGTYACAGAACAATCAG	267	2nd round	
	3192	AGGRGGTGGAAGYACTTCATTTGA			
RT-qPCR	HAV68	TCACCGCCGTTTGCCTAG	-	-	[21]
	HAV240	GGAGAGCCCTGGAAGAAAG			
	HAV150	FAM-CCTGAACCTGCAGGAATTAA-MGBNFQ			
	Mengo110	GCGGGTCCTGCCGAAAGT	-	-	[25]
	Mengo209	GAAGTAACATATAGACAGACGCACAC			
	Mengo147	FAM-ATCACATTACTGGCCGAAGC-MGBNFQ			

**Table 2:** Distribution of Hepatitis A Virus (HAV)-positive samples and of genotypes/variants according to year and source of isolation.

Period	Type of Sample (Suffix)	N° of Samples	N° of Positive Samples	Genotype (N° of Sequences and Clusters)
2015	Sewage (WTP)	30	4	IA (3 cluster D)—IB (1)
	Discharge Water (DW)	29	4	IA (3 cluster D)—IB (1)
	Seawater (SW)	39	5	IA (4 cluster D)—IB (1)
	Bivalve shellfish (BV)	485	79 *	IA (2 cluster A; 7 cluster D)—IB (2)
2016–2018	Sewage (WTP)	41	16	IA (7 cluster A; 8 cluster B)—IB (1)
	Discharge Water (DW)	324	10	IA (3 cluster A; 3 cluster B)—IB (4)
	Bivalve shellfish (BV)	261	23 *	IA (10 cluster A)—IB (1)
Total		1209	141	IA (50) **—IB (11)

* In bivalve shellfish, amplification by nested PCR and characterization by sequencing was achieved only for 22 samples. ** Clusters were as follows: 22 cluster A, 11 cluster B, 17 cluster D.

## Data Availability

The data presented in this study are openly available in GenBank; accession numbers are included in the text.
